# Bacterial Over-Expression and Purification of the 3′phosphoadenosine 5′phosphosulfate (PAPS) Reductase Domain of Human FAD Synthase: Functional Characterization and Homology Modeling

**DOI:** 10.3390/ijms131216880

**Published:** 2012-12-11

**Authors:** Angelica Miccolis, Michele Galluccio, Teresa Anna Giancaspero, Cesare Indiveri, Maria Barile

**Affiliations:** 1 Department of Biosciences, Biotechnology and Biopharmaceutics, University of Bari “A. Moro”, via Orabona 4, I-70126, Bari, Italy; E-Mail: angelica.miccolis@uniba.it; 2 Department of Cellular Biology, University of Calabria, via Bucci 4c, I-87036, Arcavacata di Rende, Italy; E-Mails: mgalluccio@unical.it (M.G.); indiveri@unical.it (C.I.); 3 Institute of Biomembranes and Bioenergetics, CNR, via Amendola 165/A, I-70126, Bari, Italy; E-Mail: teresaanna.giancaspero@uniba.it

**Keywords:** FLAD1, human FAD synthase, FMN adenylyltransferase, Flavin, FAD, PAPS reductase domain, molybdopterin-binding domain

## Abstract

FAD synthase (FADS, EC 2.7.7.2) is a key enzyme in the metabolic pathway that converts riboflavin into the redox cofactor, FAD. Human FADS is organized in two domains: -the 3′phosphoadenosine 5′phosphosulfate (PAPS) reductase domain, similar to yeast Fad1p, at the *C*-terminus, and -the resembling molybdopterin-binding domain at the *N*-terminus. To understand whether the PAPS reductase domain of hFADS is sufficient to catalyze FAD synthesis, *per se*, and to investigate the role of the molybdopterin-binding domain, a soluble “truncated” form of hFADS lacking the *N*-terminal domain (Δ_1-328_-hFADS) has been over-produced and purified to homogeneity as a recombinant His-tagged protein. The recombinant Δ_1-328_-hFADS binds one mole of FAD product very tightly as the wild-type enzyme. Under turnover conditions, it catalyzes FAD assembly from ATP and FMN and, at a much lower rate, FAD pyrophosphorolytic hydrolysis. The Δ_1-328_-hFADS enzyme shows a slight, but not significant, change of *K*_m_ values (0.24 and 6.23 μM for FMN and ATP, respectively) and of *k*_cat_ (4.2 × 10^−2^ s^−1^) compared to wild-type protein in the forward direction. These results demonstrate that the molybdopterin-binding domain is not strictly required for catalysis. Its regulatory role is discussed in light of changes in divalent cations sensitivity of the Δ_1-328_-hFADS *versus* wild-type protein.

## 1. Introduction

The primary role of the water-soluble vitamin B_2_, *i.e.*, riboflavin (Rf), in cell biology is connected with its conversion into FMN and FAD, the cofactors of a large number of dehydrogenases, reductases and oxidases involved in energetic metabolism, redox homeostasis and protein folding, as well as in diverse regulatory events [1–3]. Two enzymes are required for flavin cofactor synthesis starting from the vitamin: Rf kinase (RFK, ATP:riboflavin 5′ phosphotransferase, EC 2.7.1.26), which transfers a phosphoryl group from ATP to Rf to form FMN, and FMN adenylyl transferase (FMNAT, ATP: FMN adenylyl transferase, EC 2.7.7.2), that adenylates FMN to give FAD. Even though FMNAT is the adequate name for this enzyme, FAD synthase or, previously, synthetase (FADS) is the commonly used name.

The first eukaryotic genes coding for monofunctional RFK and FADS were identified in *Saccharomyces cerevisiae* and named *FMN1*[4] and *FAD1*[5], respectively. Fmn1p shows sequence and structure similarity to the RFK-module of prokaryotic FADS and appears largely conserved through evolution. Conversely, Fad1p, which belongs to the 3′phosphoadenosine 5′phosphosulfate (PAPS) reductase family, has little or no sequence similarity to the bacterial FADS domain [6,7]. Its structural characterization has been recently reported [8], as had that of an enzyme from the pathogenic yeast *Candida glabrata*[9]. Thus, FADS is particularly interesting as a potential target for the development of novel antimicrobial drugs [10,11].

Homology search using Fad1p as a template led to the identification of the human gene for FADS, named *FLAD1*, which is localized on chromosome 1 [12,13]. Two isoforms, namely isoforms 1 and 2, have been identified by enzymatic assay and mass spectroscopy [12,13]. hFADS1 is a 587-amino acid protein with a predicted molecular mass of 65.3 kDa; hFADS2 is a 490-amino acid protein with a predicted molecular mass of 54.2 kDa, which lacks a 97 amino acid segment in the *N*-terminal region of hFADS1. The first 17 residues (*i.e.*, 2.1 kDa) of hFADS1 represent a putative mitochondrial targeting peptide as predicted by bioinformatic analysis [12]. The mitochondrial localization of hFADS1, together with the cytosolic localization for hFADS2, was then experimentally demonstrated in [14].

The human cytosolic FADS, hFADS2, was produced and purified to homogeneity as a recombinant His-tagged protein in [15]. The enzyme binds one mole of the FAD product very tightly, although non-covalently, with a FAD/protein ratio equal to 0.86 ± 0.2 mol FAD per hFADS2 monomer. Since newly synthesized FAD is expected to be delivered to “client” apoflavoprotein, a question remains about the structural requirements for FAD release. Human FADS structure has yet to be resolved by X-ray crystallography. At the moment, other FADS isoforms of different length, at present, are reported in the Ensembl database (http://www.ensembl.org/index.html); for better detail, see [16].

In humans (and, in general, in most higher eukaryotes), FADS differs from its yeast counterpart as being organized in two domains. Besides the PAPS reductase domain (InterPro database accession number: IPR002500), it contains an additional domain, localized at the *N*-terminus of the human enzyme, which resembles a molybdopterin-binding domain (InterPro database accession number: IPR012183). This domain is similar to *Arabidopsis thaliana* Cnx1G (23.7% identity, 35.3% similarity), *Drosophila melanogaster* cinnamon protein (22.6% identity, 35.1% similarity) and human gephyrin (16.8% identity, 30.6% similarity). This domain is also present in the *Escherichia coli* monofunctional mogA (25.6% identity, 35.9% similarity) and moaB (22.9% identity, 37.1% similarity) proteins, which are involved in molybdenum cofactor biosynthesis, the prosthetic group of molybdoenzymes, such as sulfite oxidase and xanthine oxidase (see [17,18]). The function of this domain in hFADS remains completely unknown.

The human PAPS reductase domain of hFADSs shares 34% identity and 60% similarity with the corresponding yeast Fad1p domain. Thus, here we studied whether, as for yeast, the PAPS reductase domain of the hFADS is sufficient to catalyze FAD synthesis, *per se*. Furthermore, we investigated the possible role of the *N*-terminus resembling molybdopterin-binding domain on catalytic activity.

## 2. Results and Discussion

### 2.1. Homology Modeling of the PAPS Reductase Domain of the hFADS

**Figure 1:**
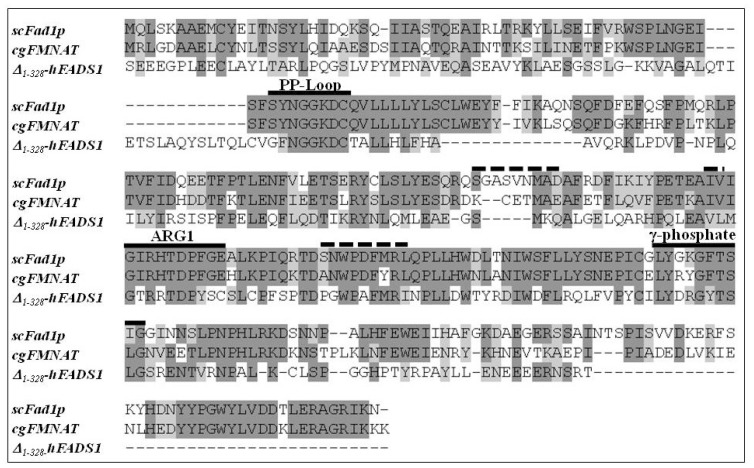
Multi-alignment of Fad1p from *Saccharomyces cerevisiae* (scFad1p), FMN adenylyltransferase from *Candida glabrata* (cgFMNAT) and the PAPS reductase domain of hFADS1 (Δ_1-328_-hFADS). The multi-alignment was performed by ClustalW2 software (European Bioinformatics Institute: Cambridge, UK). Identical amino acids are reported in dark grey, similar amino acids are reported in light grey. The amino acids involved in the conserved PP-Loop, ARG1 and γ*-*phoshate motifs (>50% identity in the three proteins) are underlined by continuous lines. The amino acids involved in Flavin motif (<50% identity in the three proteins) are underlined by dotted lines.

**Figure 2:**
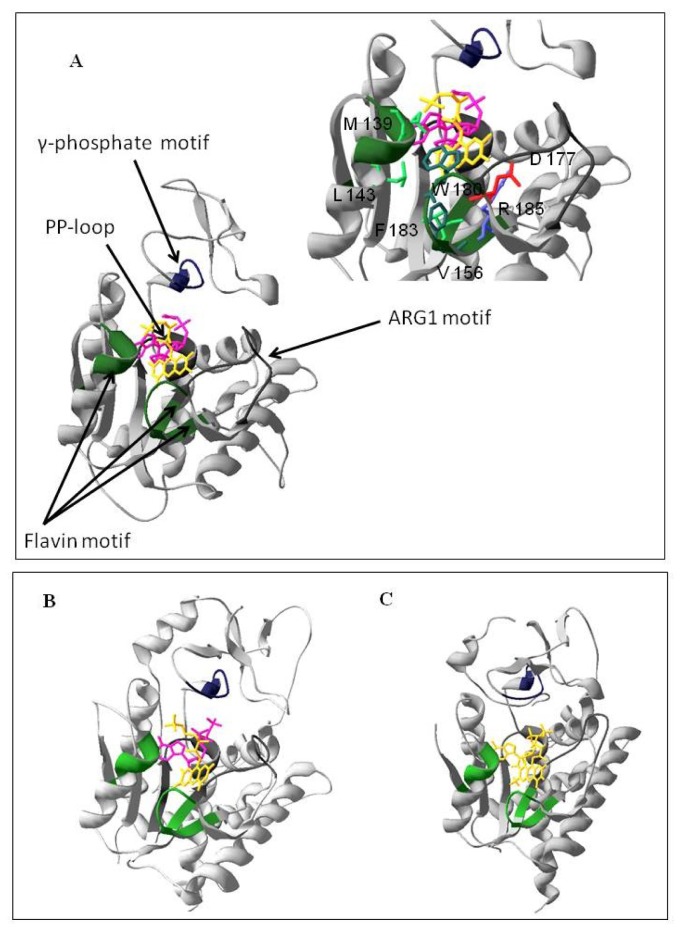
Homology structural model of the PAPS reductase domain of hFADS and view of the active site. (**A**) The model was obtained by Modeller 9.10 software (Andrej Sali, University of California: San Francisco, USA) using the FMN adenylyltransferase from *Candida glabrata* (PDB: 3G5A_A) as a template. Domains corresponding to the conserved motifs (see Figure 1) are highlighted by colors and indicated by arrows. The substrates FMN (yellow) and AMPCPP (pink) have been docked using ArgusLab software (Planaria Software LLC: Lake Forest Park, USA). Amino acids involved in substrate binding are indicated in the right side of the figure; (**B**) The crystal structure of *Candida glabrata* FMN Adenylyltransferase (PDB: 3G5A_A); (**C**) The crystal structure of yeast FAD synthetase (FAD1) (PDB: 2WSI).

To identify which of the two domains of hFADS is responsible for FAD synthesis starting from FMN, a multi-alignment was performed among the hFADS1 (defined as the “canonical” sequence by UniprotKB), FMN adenylyltransferase from *C. glabrata* and Fad1p from *S. cerevisiae*. The alignment showed a consensus sequence of 64 amino acids (corresponding to 24% of the sequences lengths) among the 329–587 region of hFADS1 and the other two proteins (Figure 1). The most conserved regions of the proteins, which should be important for the catalysis, correspond to the PP-loop, the ARG1 loop, the flavin and the γ-phosphate motifs (Figure 1). On the basis of the 28% pair-wise identity, the homology model of the PAPS reductase domain was built using as a template the FMNAT of *C. glabrata* (PDB code: 3G5A) (Figure 2). The model was geometrically validated by *MolProbity* web service (MolProbity score: 2.98) [19]. In this structure the PP-loop, the ARG1 loop, the flavin and the γ-phosphate motifs, which correspond to the conserved region, are highlighted. The predicted location of FMN and AMPCPP and the relationships with the enzyme are shown. The isoalloxazine ring is in the middle of the predicted flavin motif, which is composed largely of loops (64%) interlaced by α6–α9 helices and two short 3_10_-helices. The folding of the PAPS reductase domain resembles those of the related enzymes FMNAT of *C. glabrata* and Fad1p from *S. cerevisiae* (Figure 2B,C), in agreement with the rmsd value of 1.10 Å for 163 superimposed *C*α atoms of the PAPS reductase domain (from Q70 to K232) on FMNAT of *C. glabrata*, calculated by Swiss-PdbViewer 4.0.1. (Swiss Institute of Bioinformatics: Lausanne, Switzerland). The structure has a prevailing α/β fold with central β-sheets surrounded by α-helices, and the region containing the active site seems to be conserved in the three enzyme structures as it appears from the comparison of Figure 2A with Figure 2B,C. The small segment containing the γ-phosphate motif has a higher random coil content, thus appearing less similar to the related enzymes of *C. glabrata* and *S. cerevisiae*. In analogy to the FMNAT of *C. glabrata*[9], several residues are involved in the interaction with the isoalloxazine ring, including the aromatic W180 and F183, the hydrophobic M139, L143 and V156 and the hydrophilic D177 and R185 (inset to Figure 2A).

### 2.2. Expression of the Δ_1-328_-hFADS and Purification as a FAD-Binding Domain

**Figure 3:**
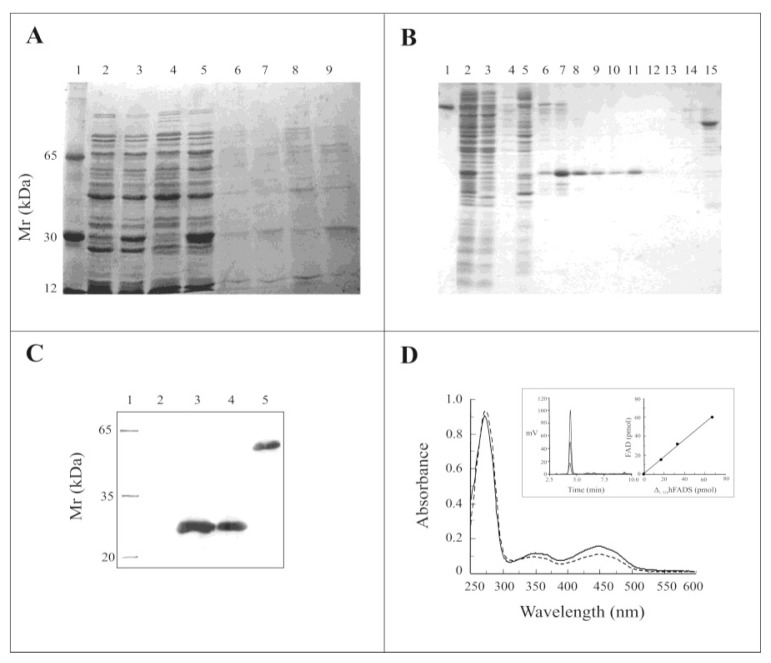
Expression, purification and spectroscopic properties of recombinant 6-His-Δ_1-328_-hFADS. (**A**) Proteins were separated by SDS-PAGE on a 15% gel and stained with Coomassie Blue. Lane 1, molecular weight markers; lane 2, un-induced supernatant after 2 h growth; lane 3, IPTG-induced supernatant after 2 h growth; lane 4, un-induced supernatant after 4h growth; lane 5, IPTG-induced supernatant after 4h growth; lanes 6–9, corresponding insoluble fractions of the sample of lanes 2–5; (**B**) Protein fractions obtained by Ni^2+^-chelating chromatography were separated by SDS-PAGE on a 15% gel and stained with Coomassie Blue. Lane 1, BSA (2 μg); lane 2, IPTG-induced supernatant (20 μg); lane 3, first flow through fraction (19.8 μg); lane 4, second flow through fraction (4 μg); lane 5, proteins eluted with 50 mM imidazole (27.7 μg); lane 6, first fraction of proteins eluted with 150 mM imidazole (0.8 μg); lane 7, second fraction of proteins eluted with 150 mM imidazole (4 μg); lane 8, third fraction of proteins eluted with 150 mM imidazole (2.5 μg); lane 9, fourth fraction of proteins eluted with 150 mM imidazole (1.5 μg); lane 10, first fraction of proteins eluted with 400 mM imidazole (1.3 μg); lane 11, second fraction of proteins eluted with 400 mM imidazole (2.4 μg); lane 12, third fraction of proteins eluted with 400 mM imidazole (0.3 μg); lane 13, fourth fraction of proteins eluted with 400 mM imidazole (0.3 μg); lane 14, fraction of proteins eluted with 500 mM imidazole (0.3 μg); lane 15, fraction of the 6-His-hFADS2 eluted with 400 mM imidazole (2.6 μg); (**C**) Analysis by immunoblotting of purified 6-His-Δ_1-328_-hFADS. Lane 1, molecular mass marker; lane 2, BSA (2 μg); lane 3, fraction 7 of (B); lane 4, fraction 11 of (B); lane 5, 6-His-hFADS2; (**D**) The spectra of either 6-His-Δ_1-328_-hFADS (continuous line, 15.9 μM) or 6-His-hFADS2 (dashed line, 11.7 μM) were recorded in 40 mM Hepes/Na, 5 mM β-mercaptoethanol, pH 7.4. In the inset, the purified 6-His-Δ_1-328_-hFADS (3.8 μM) was treated with 10% perchloric acid. The supernatant was neutralized, and aliquots were analyzed by HPLC for flavin content and reported *versus* protein monomer amount.

The recombinant pH6EX3-Δ_1-328_-hFADS construct, encoding the PAPS reductase domain of the hFADS1 fused with the extra *N*-terminal sequence MSPIH_6_LVPRGSEASNS, was used for heterologous expression in the *E. coli* Rosetta (DE3) strain. The expression of the Δ_1-328_-hFADS protein was performed starting from conditions previously optimized for hFADS2 [13]. Optimal expression was obtained 12 h after induction with 0.5 mM isopropyl-thio-β-d-galactoside (IPTG) at 20 °C. Electrophoresis of the IPTG-induced and un-induced cell lysates showed a heavily stained protein band mostly present (more than 70%) in the soluble fraction of the induced cell lysate (Figure 3A, lane 3, 5), which was absent in the corresponding soluble fractions of the non-induced cell lysate (Figure 3A, lanes 2 and 4). A little amount of protein was present in the IPTG induced insoluble fractions (Figure 3A, lanes 7 and 9). The over-expressed protein migrated on SDS-PAGE at a calculated molecular mass of about 28 kDa, not far from the theoretical molecular mass of the recombinant 6-His-Δ_1-328_-hFADS (31499 Da) calculated by the Expasy tool Compute pI/MW at the web site http://www.expasy.org. The soluble fraction, corresponding to lane 5 of Figure 3A, was applied on Chelating Sepharose fast flow resin and, after washing, eluted with a step gradient of 50 mM, 150 mM, 400 mM and 500 mM imidazole. Purified fractions, eluted with imidazole 400 mM, showed on SDS-PAGE a single protein band of about 28 kDa (Figure 3B, lanes 10–13). The yield of the protein was about 22 mg/L of cell culture (average of three preparations).

The recombinant Δ_1-328_-hFADS was then identified with anti-FADS polyclonal antibody raised against recombinant hFADS2, produced according to [13] (see [14] for better details). A marked band was immuno-detected in all the fractions containing the over-expressed protein, demonstrating that it corresponded to a domain of hFADS (Figure 3C). The absorbance spectrum of the recombinant Δ_1-328_-hFADS (Figure 3D, straight line) showed a typical flavoprotein absorbance spectrum, similar to that of the entire hFADS2 [15], with a main peak at 275 nm and two minor peaks at ~350 and 450 nm. The FAD/protein monomer ratio estimated in four different protein preparations from the absorption spectrum (Fl%), as described in Section 3.7, was equal to 0.82 ± 0.13, in good agreement with the FAD/protein monomer ratio estimated for the wild-type protein in [15]. Precipitation of the purified holoprotein with trichloroacetic acid resulted in complete removal of the flavin (data not shown). In an alternative procedure, HPLC analysis of the supernatant obtained by acidic treatment of purified Δ_1-328_-hFADS demonstrates that the bound cofactor is FAD with a FAD/protein monomer ratio corresponding to that measured from the absorption spectrum (inset to Figure 3D).

### 2.3. Biochemical and Functional Characterization of 6-His-Δ_1-328_-hFADS

**Figure 4:**
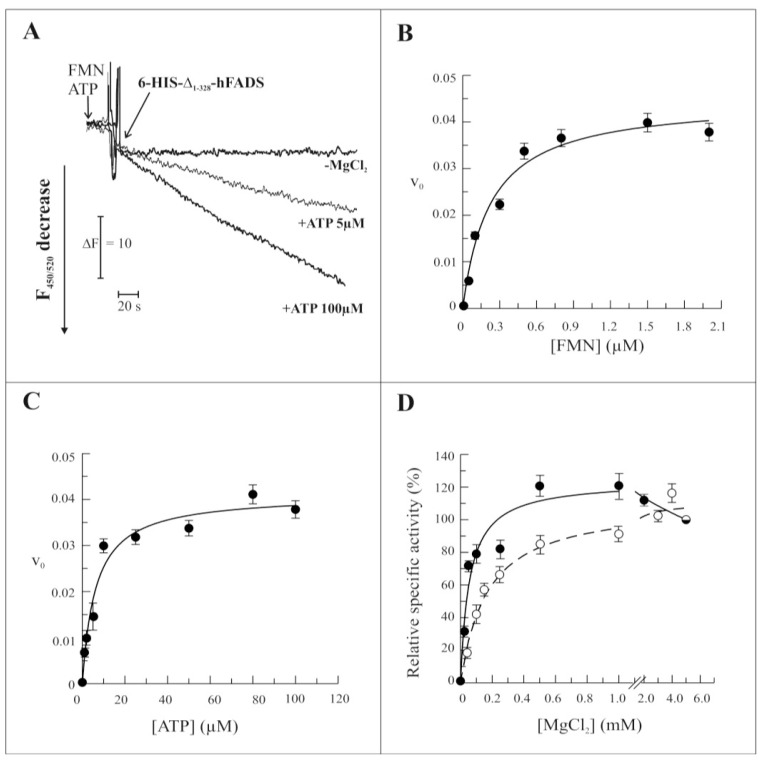
Functional and kinetic characterization of FAD synthesis catalyzed by 6-His-Δ_1-328_-hFADS. (**A**) The purified 6-His-Δ_1-328_-hFADS (0.32 nmol) was incubated at 37 °C in 50 mM Tris/HCl pH 7.5 containing 2 μM FMN and ATP at the reported concentrations, in the presence or absence of 5 mM MgCl_2_. The FAD synthesis reaction was started by the addition of recombinant protein, and its v_0_ was measured by the initial rate of fluorescence decrease (excitation at 450 nm, emission at 520 nm) and expressed as μmol FMN · s^−1^ · μmol Δ_1-328_-hFADS^−1^; (**B**,**C**) FAD synthesis rate, catalyzed by purified 6-His-Δ_1-328_-hF ADS (●, 0.32 nmol), was measured as dependence of the indicated FMN (B) and ATP (C) concentrations, in the same experimental condition described in (A). Data points are fitted according to the Michaelis-Menten equation, as described in the Experimental Section; (**D**) The ability of the purified 6-His-Δ_1-328_-hFADS (●, 0.32 nmol) to synthesize FAD is plotted as a function of MgCl_2_ concentration in the experimental conditions described in (A). The FAD synthesis rate catalyzed by 6-His-hFADS2 (○, 0.17 nmol) is reported as control. Data, normalized to the activity measured in the presence of 5 mM MgCl_2_ for each protein (arbitrarily set equal to 100%), are fitted according to the Michaelis-Menten equation, as described in Experimental Section.

The ability of the recombinant Δ_1-328_-hFADS to catalyze FAD synthesis was demonstrated and the rate of reaction measured by following flavin fluorescence decrease in the presence of the substrates FMN and ATP, essentially as in [15]. As expected, the reaction rate depended on the ATP concentration, being more than double at 100 μM with respect to 5 μM ATP (Figure 4A). As in the case of the entire hFADS2 and of the yeast enzyme [20], the recombinant Δ_1-328_-hFADS required the presence of MgCl_2_ (Figure 4A). The kinetics of the reaction were studied as a function of the concentration of FMN, ATP and MgCl_2_ (Figure 4, panels B–D). The dependence of the reaction rate on FMN and ATP concentrations showed saturable behaviors similar to those previously found for the wild-type protein [15], and the experimental data could be fitted by the Michaelis–Menten equation with half saturation constants (*K*_m_) of 0.24 ± 0.06 μM and 6.23 ± 1.5 μM for FMN and ATP, respectively, and with a *k*_cat_ value of 0.041 s^−1^ (Figure 4B,C). An average *K*_cat_ value of 0.042 ± 0.001 s^−1^ was calculated from four experiments, performed with different protein preparations. The dependence of the reaction rate on the concentration of MgCl_2_ of Δ_1-328_-hFADS showed an increased sensitivity towards the divalent cation to the wild-type protein (dashed line), with 50% of maximum activity (Mg^2+^_50_) at 0.05 mM and maximal activity at 0.5–1.0 mM. The distinctive feature of the recombinant Δ_1-328_-hFADS was a decrease of synthesis rate at higher MgCl_2_ concentrations (Figure 4D).

**Figure 5:**
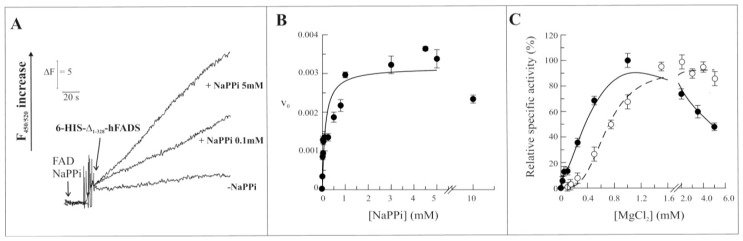
Functional and kinetic characterization of FAD cleavage catalyzed by 6-His-Δ_1-328_-hFADS. (**A**) The purified 6-His-Δ_1-328_-hFADS (0.32 nmol) was incubated at 37 °C in 50 mM Tris/HCl pH 7.5 containing 0.5 μM FAD, 5mM MgCl_2_ and in the absence or presence of NaPPi at the reported concentrations. The FAD cleavage reaction was started by the addition of 6-His-Δ_1-328_-hFADS, and its v_0_ was measured by the initial rate of fluorescence increase (excitation at 450 nm, emission at 520 nm) and expressed as μmol FAD · s^−1^ · μmol Δ_1-328_-hFADS^−1^; (**B**) The FAD cleavage rate, catalyzed by purified 6-His-Δ_1-328_-hFADS (0.32 nmol), was measured as a dependence of the indicated NaPPi concentrations in the same experimental condition described in (A). Data points are fitted according to the Michaelis-Menten equation, as described in Experimental Section; (**C**) The ability of the purified 6-His-Δ_1-328_-hFADS (●, 0.32 nmol) to catalyze the reverse reaction is plotted as a function of MgCl_2_ concentration in the experimental conditions described in (A). The FAD synthesis rate catalyzed by 6-His-hFADS2 (○, 0.17 nmol) is reported as control. Data are normalized to the maximum activity measured for each protein (arbitrarily set equal to 100%).

As in the case of the entire hFADS2 [15], the recombinant Δ_1-328_-hFADS is able to catalyze the reverse reaction. The pyrophosphorolitic activity was also measured in the presence of the substrates FAD and NaPPi. An increase of fluorescence was detected as due to the formation of FMN (Figure 5A). The dependence of FAD pyrophosphorolytic rate on NaPPi concentration was studied, and also in this case, data could be fitted in the Michaelis-Menten equation with a *K*_m_ of 92.0 ± 0.5 μM and with a *k*_cat_ value of 0.0032 s^−1^ (Figure 5B). An average *K*_cat_ value of 0.0037 ± 0.0009 s^−1^ was calculated from four experiments, performed with different protein preparations. At concentrations of the substrate above 4 mM, the activity slightly decreased to a value corresponding to about 80% of the maximal rate at 10 mM NaPPi. Similarly to the forward reaction, the dependence of the pyrophosphorolytic rate on the MgCl_2_ concentration (Figure 5C) catalyzed by the recombinant Δ_1-328_-hFADS (continuous line) was sensibly different from the entire hFADS2 (dashed line). In this case the sigmoidal shape of the Mg^2+^ dependence of the wild-type protein was transformed in a hyperbolic one with Mg^2+^_50_ of about 0.2 mM. Moreover, the rate of the reverse reaction, catalyzed by the recombinant Δ_1-328_-hFADS, decreased at MgCl_2_ concentrations above 1 mM. The reduction of reaction rate was even more evident than in the forward reaction with an activity at 5 mM MgCl_2_ of about half with respect to the maximal one, at 1 mM MgCl_2_.

**Figure 6:**
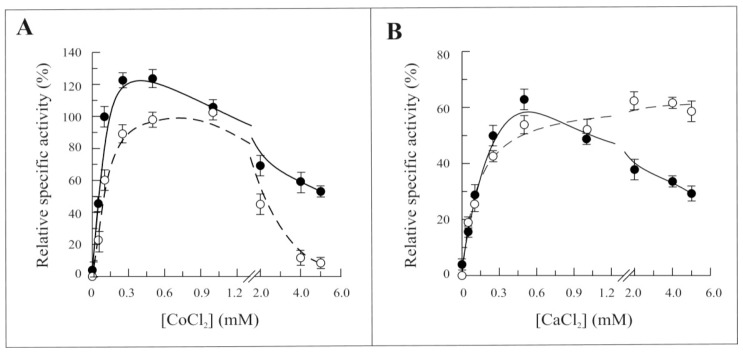
Dependence of the 6-His-Δ_1-328_-hFADS activity on CoCl_2_ and CaCl_2_ concentrations. The ability of 6-His-Δ_1-328_-hFADS (●, 0.32 nmol) and 6-His-hFADS2 (○, 0.17 nmol) to synthesize FAD is plotted as a function of CoCl_2_ (**A**) or CaCl_2_ (**B**) concentrations in the presence of 100 μM ATP and 2 μM FMN and in the absence of MgCl_2_ in the same experimental condition described in Figure 4A. Data are normalized to the maximum activity measured with 5 mM MgCl_2_ for each protein (arbitrarily set equal to 100%).

The dependence of the FAD synthesis reaction rate on the concentration of CoCl_2_ or CaCl_2_ was studied in the absence of MgCl_2_ (Figure 6A,B, respectively). Both Co^2+^ and Ca^2+^ were able to substitute Mg^2+^, Co^2+^ being more effective than Ca^2+^. The dependence of the reaction rate on the concentration of both these cations resembled that of MgCl_2_ up to about 0.5 mM concentration, with an increased sensitivity towards the Co^2+^ shown by Δ_1-328_-hFADS. At higher concentrations of CoCl_2_, the activity drastically decreased to less than 50% of the maximal for the recombinant Δ_1-328_-hFADS, while for the entire hFADS2, the activity was completely abolished. A similar decrease in the reaction rate was observed with CaCl_2_ for the recombinant Δ_1-328_-hFADS (continuous line), but not for the wild-type enzyme (dashed lines).

**Figure 7:**
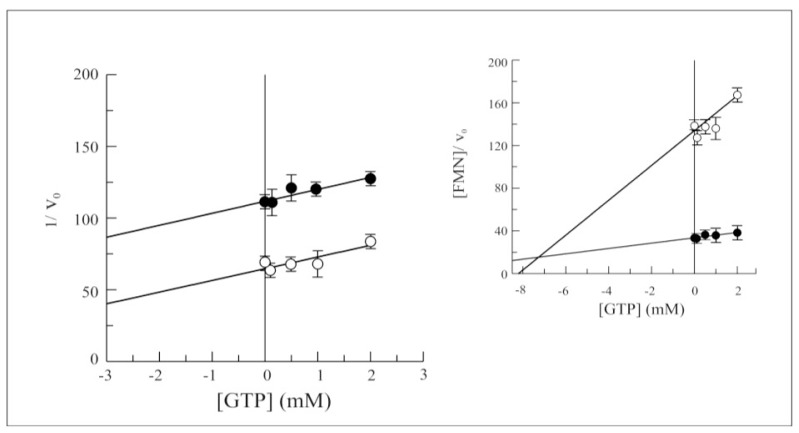
GTP inhibition on FAD synthesis catalyzed by 6-His-Δ_1-328_-hFADS. FAD synthesis rate catalyzed by purified 6-His-Δ_1-328_-hFADS (0.32 nmol) was fluorimetrically measured at 37 °C in 50 mM Tris/HCl pH 7.5 containing 5 mM MgCl_2_, 100 μM ATP and FMN (0.3 μM, ○ or 2 μM,●). GTP was added in the FAD synthesis reaction mixture at the indicated concentrations. In the inset, the plot of the FMN concentration/FAD synthesis rate ratio *vs.* GTP concentration is reported to estimate *K*_i_ value.

As previously observed for the wild-type protein [15], the purine nucleotides GTP and GDP were found to inhibit FAD synthesis rate. The effect of both these nucleotides was tested on the rate of the forward reaction catalyzed by Δ_1-328_-hFADS. While no significant changes were found in competitive inhibition by GDP (data not shown), the Dixon plot reported in (Figure 7) demonstrated that Δ_1-328_-hFADS is less sensitive towards the uncompetitive inhibition by GTP, with a *K*_i_ value equal to 7.5 mM (see inset to Figure 7), *i.e.*, four times higher than that measured for the wild-type enzyme.

In Table I, a comparison between the kinetic properties of the truncated Δ_1-328_-hFADS (this study) and the entire protein 6-His-hFADS2 [15] is reported.

### 2.4. Discussion

The identification of human enzyme responsible for synthesis of FAD, coded by the gene FLAD1, was achieved some years ago by homology searching using the product of *S. cerevisiae FAD1* gene as a template [12]. It is noteworthy that, differently from the yeasts enzyme consisting of a single structural domain (PAPS reductase, IPR002500), the FADSs from mammals are organized in two domains. This article deals with the structural and functional characterization of the PAPS reductase domain present at the *C*-terminus of isoform 1 and 2 of the human enzyme.

It is not surprising that while the Fad1p of *S. cerevisiae* and the FMNAT of *C. glabrata* are very similar to each other, the human PAPS reductase domain shows much less identity with the enzymes of the lower organisms. However, some of the important structural features of the enzymes are well conserved, also in the human one, such as the PP-loop and ARG1, which are involved in ATP binding, the flavin motif, that is involved in FMN binding and the γ-phosphate motif, whose role is still not clear [9]. The structure of the FADS of higher organisms is still unknown, as well as that of the human PAPS reductase domain, which has been designed and expressed for the first time in this work. Thus, the homology modeling strategy revealed some interesting properties of the recombinant Δ_1-328_-hFADS. Indeed, the tertiary structure and, hence, the folding of the enzyme may be better conserved than the primary structure as it appears from the comparison of homology structural model of the human PAPS reductase domain with the structure of the lower organism enzymes [8,9]. These observations, however, need to be proven by the X-ray structure.

Here, we report the characterization of a His-tagged recombinant polypeptide, namely Δ_1-328_-hFADS, corresponding to the PAPS reductase domain of hFADS, which was purified in a holo-form containing FAD tightly bound.

Experiments of functional characterization let us conclude that the PAPS reductase domain, *per se*, is able to catalyze the typical reactions of the FADS: FAD synthesis and its cleavage. The turnover number of Δ_1-328_-hFADS (*k*_cat_ 4.2 × 10^−2^ s^−1^) is even lower than that measured for the entire enzyme, thus leaving open the hypothesis that FAD release may represent the rate-limiting step of the whole catalytic cycle of FAD synthesis process [15]. Accordingly, the percentage of flavinylation of the protein, which might be related with the enzyme efficiency, is not significantly varied between hFADS and the truncated form. Nevertheless, some properties concerning the response to Mg^2+^, Co^2+^ and Ca^2+^ and also to purine nucleotide GTP are quite different from those of the entire human enzyme. These diversities indicate that the lack of the molybdopterin-binding domain results in regulatory differences and, hence, it should play some roles in the regulation of the enzyme hFADS.

The truncated form resulted in being more sensitive to divalent ions at low concentrations. We could postulate that the *N*-terminus molybdopterin-binding resembling domain somehow hid the binding of the ions and, hence, of the substrates at the PAPS active site. On the other hand, during the reverse reaction, the *N*-terminus domain could participate in modulating some Mg^2+^-dependent conformational changes, accompanying the pyrophosphorolytic disassembly of FAD cofactor, presumably binding this ion in a different site. The increase of the inhibitory effect by Mg^2+^ and Ca^2+^ at high concentrations may be the consequence of their interaction with negatively charged residues of the protein available in high number, as revealed by the calculation of the molecular surface of the protein, leading to protein instability. This phenomenon may not occur or be limited in the entire enzyme by complete or partial occlusion of these interaction sites by the presence of the molybdopterin-binding domain.

Based on the uncompetitive type of inhibition and on the different *K*_i_ between the entire and the truncated proteins, we postulate that the *N*-terminus domain resembling a molybdopterin-binding domain in the human FADSs may contribute to allocating GTP in a site different from the substrate sites. The ability of the *N*-terminus molydbopterin-binding domain to bind ions Mg^2+^ and purine nucleotides is not surprising, since the functional role of this domain in mogA and moaB proteins is to catalyse, in a Mg^2+^ and ATP-dependent way, the adenylation of molybdopterin [21]. The Cnx1G domain in the *A. thaliana* Cnx1 performs essentially the same function and is also responsible for copper coordination to molybdopterin-dithiolate sulphurs; its crystal structure has been solved in its apo-form (PDB: 1UUX) and in complex with molybdopterin and adenylated-molybdopterin (PDB: 1UUY) [22]. The *Drosophila* cinnamon gene, too, is functional homologous to the Arabidopsis Cnx1 [23].

In humans the multifunctional protein gephyrin is thought to catalyze the final step in molybdenum biosynthesis, *i.e.*, the covalent insertion of a molybdate ion into molybdopterin, with the molybdopterin binding domain located in the *N*-terminus part of the protein, performing an adenilyl transferase activity [24]. However, additional and various functional roles have been proposed for gephyrin [24] and for cinnamon [23], thus leaving open the question of the actual function of this domain in different protein contexts. The multidomain protein gephyrin was initially discovered in the central nervous system to organize the glycine receptor, GlyR, within the postsynaptic membrane and later on in non-neuronal tissues. Alternative splicing has been proposed to contribute to gephyrin’s functional diversity within single cells, as well as in different cell types and tissues.

The function of the *N*-terminus domain, resembling a molybdopterin-binding domain, present in mammal FADSs, still remains to be investigated in further detail. We do not known whether and how the full-length hFADS protein is able to catalyse adenylation of molybdopterin, and at the moment, we have no idea about the structural relationship between this domain with the PAPS reductase domain in the hFADSs, but the ability to modulate GTP and divalent ions response introduces a novel role for molybdopterin-binding domain in the regulation of flavin cofactor biosynthesis.

Finally, we would like to point out that the *N*-terminus portion of this domain generated by alternative splicing of the *FLAD1* gene (*i.e.*, the first 97 amino acids) differs in hFADS1 with respect to hFADS2, and it is surely involved in protein sub-cellular localization [14]. This aspect, together with the sub-cellular localization of the different FADS isoforms in human cells [16], is a matter of future investigation in our laboratory.

Since FAD-forming enzymes are strictly required for microbial viability [10,16], and the human FADSs are unrelated to those of prokaryotic FADSs, and also somehow different from that of pathogenic microorganisms [10,11], the structural and functional data on the recombinant Δ_1-328_-hFADS may constitute a tool for high-throughput screening of toxicity of specific inhibitors/modulators that inactivate the microbial FAD-forming enzymes, but not the human ones.

## 3. Experimental Section

### 3.1. Materials

All chemicals were from Sigma-Aldrich (St. Louis, MO, USA), unless otherwise specified. The *Escherichia coli* Rosetta (DE3) strain was purchased from Novagen (Madison, WI, USA). Restriction endonucleases and other cloning reagents were purchased from Fermentas (Glen Burnie, MD, USA). Bacto Peptone and Bacto Yeast extract were from Difco (Lawrence, KS, USA). Chelating Sepharose Fast Flow was from Amersham Biosciences (Arlington Heights, IL, USA). The dye reagent for protein assay was from Bio-Rad (Hemel Hempstead, Herts, UK).

### 3.2. Identification of PAPS Reductase Domain of the Human FAD Synthase

The PAPS reductase domain of the hFADS1 (amino acid sequence 329–587) was identified by multi-alignment among FMN adenylyltransferase from *C. glabrata*, Fad1p of *S. cerevisiae* and hFADS1 by using ClustalW2 (EBI-EMBL tool, www.ebi.ac.uk/Tools/msa/clustalw2/). The consensus sequence represented 24% of the 329–587 region of hFADS1.

### 3.3. Homology Modeling of the Human PAPS Reductase Domain

The identified human PAPS reductase domain sequence was modeled by Modeller 9.10 software (Andrej Sali, University of California: San Francisco, USA) using the FMN adenylyltransferase from *C. glabrata* (PDB: 3G5A_A) as template [9,25].

### 3.4. Cloning of PAPS Reductase Domain of the Human FAD Synthase

To clone the PAPS reductase domain of the hFADS1, the corresponding region was amplified using the forward and reverse primers 5′-CCGGAATTCATCAGAGGAAGAAGGACCCCT-3′ and 5′-GACCCTCGAGTCATGTGCGGGAGTT-3′, containing the *Eco*RI and *Xho*I sites, respectively. The amplified DNA was cloned in the *EcoR*I/*Xho*I sites of the pH6EX3 expression vector. The resulting recombinant plasmid, defined as pH6EX3-Δ_1-328_-hFADS, carried the extra *N*-terminal sequence MSPIHHHHHHLVPRGSEASNS.

### 3.5. Expression of the PAPS Reductase Domain of the Human FAD Synthase in *E. coli*

Rosetta(DE3) strain was transformed with the pH6EX3-Δ_1-328_-hFADS plasmid by calcium chloride treatment. Selection of transformed colonies was performed on LB-agar plates containing 100 μg/mL ampicillin and 34 μg/mL chloramphenicol. *E. coli* Rosetta (DE3) cells carrying the recombinant plasmids were inoculated in 10 mL of LB medium (1% Bacto Peptone, 0.5% Bacto Yeast extract, 1% NaCl, pH 7.0) supplemented with 100 μg/mL ampicillin and 34 μg/mL chloramphenicol, and cultured overnight at 37 °C with rotary shaking (≅200 rpm). A 5 mL-aliquot of the cell culture was transferred to 0.5 L of fresh LB medium supplemented with 100 μg/mL ampicillin and 34 μg/mL chloramphenicol and grown at 37 °C to A_600_ equal to 0.6–0.7. Then, 0.5 mM IPTG was added to induce the expression of the recombinant protein, 6-His-Δ_1-328_-hFADS. Growth was continued overnight at 20 °C, bacteria were harvested by centrifugation at 3000 *g* for 10 min at 4 °C and the pellets stored at −20 °C. The bacterial pellet (about 3 g wet weight) was thawed on ice for 15 min and resuspended in 30 mL start buffer (500 mM NaCl, 40 mM Hepes/Na, pH 7.4) supplemented with 0.2 mL of Protease Inhibitor Cocktail (P8849, Sigma-Aldrich) and 0.5 mM PMSF. Cells were disrupted by mild sonication at 4 °C (three pulses for 60 s, 30 s and 30 s at 100 W with 60 s intermission) using a Branson Sonifier 250. The soluble and the insoluble cell fractions were separated by centrifugation of the cell lysate at 20,000*g* for 30 min at 4 °C. The pellet (inclusion bodies and cell debris), containing the insoluble over-expressed proteins, was re-suspended in 15 mL start buffer, aliquoted and used for SDS-PAGE analysis and FADS activity assay. The supernatant, containing the soluble over-expressed 6-His-Δ_1-328_-hFADS, was used for SDS-PAGE analysis, FADS activity assay and further protein purification (see below).

### 3.6. Purification of Recombinant 6-His-Δ_1-328_-hFADS of the Human FAD Synthase

A 30 mL-aliquot of the soluble cell fraction, obtained from *E. coli* Rosetta (DE3) strain transformed with the pH6EX3-Δ_1-328_-hFADS plasmid, was applied onto a Chelating Sepharose Fast Flow column (3 mL packed resin), previously charged with 250 mM NiSO_4_ according to the producer’s protocol, and equilibrated with the start buffer. The column was first washed with 30 mL start buffer, then developed with a step gradient of 50 mM, 150 mM, 400 mM and 500 mM imidazole in the same buffer. At each step of the purification procedure, the FADS activity was measured (see below) and the purity of the 6-His-Δ_1-328_-hFADS was checked by SDS-PAGE. Prior to storing or further processing, fractions containing the purified recombinant protein were desalted by gel filtration on a PD10-column in 40 mM Hepes/Na, 5 mM β-mercaptoethanol, pH 7.4. These protein samples were stable for at least 30 days at 4 °C.

### 3.7. Protein Concentration and FAD/Protein Monomer Ratio Measurements

Protein concentration was measured with the method of Bradford, using BSA as a standard [26]. In an alternative procedure, protein concentration of the purified 6-His-Δ_1-328_-hFADS was estimated by absorbance spectra, which were recorded on an Ultrospec 3100pro spectrophotometer (Amersham Biosciences), essentially as in [15]. To this aim, the contribution of the bound FAD had to be subtracted from the A_280_ readings. Because A_280_ for both free FAD and Δ_1-328_-hFADS-bound FAD is 1.7-fold A_450_, the A_280_, actually due to the apo-protein, may be calculated from the Equation:

$$A_{280\;\text{apo}-\text{enzyme}}=A_{280}-(A_{450}\times 1.7)$$

The protein concentration was then estimated by using ɛ_280_ (36,245 mM^−1^ cm^−1^, 1.151 mg/mL^−1^ cm^−1^), as calculated from the protein sequence by using the Expasy ProtParam tool (Swiss Institute of Bioinformatics: Lausanne, Switzerland). Measurements made by either the spectrophotometric or the Bradford method differ by no more than 7%.

The FAD/protein monomer ratio (given as percent flavinylation, Fl%) can be also estimated from the absorbance spectrum by considering:

$$\text{Fl}\%=[(A_{450}/A_{280\text{apo}-\text{enzyme}})/0.3]\times 100$$

where 0.3 is the ɛ_450_ FAD/ɛ_280_ apo-enzyme ratio.

### 3.8. Measurements of FAD Synthesis Rate

The rate of FAD synthesis was measured by means of the differential fluorimetric properties of FAD with respect to FMN, essentially as in [15]. Fluorescence time courses (excitation at 450 nm and emission at 520 nm) were followed at 37 °C in a LS50 Perkin Elmer spectrofluorimeter. In each experiment, FAD and FMN fluorescence were individually calibrated using standard solutions whose concentration was calculated by using ɛ _450_ = 12.2 mM^−1^ cm^−1^ for FMN and 11.3 mM^−1^ cm^−1^ for FAD. Under the experimental conditions used here, the FAD fluorescence constant (*K*_FAD_) was about 10-times lower than that of FMN (*K*_FMN_). Thus, the rate of FAD synthesis, expressed as μmol FMN · s^−1^ · μmol Δ_1-328_-hFADS^−1^, was calculated from the rate of fluorescence decrease, measured as the tangent to the initial part of the experimental curve by applying the following equation:

$$v_{0}=[(\Delta F/\Delta K)\cdot V]/t\cdot m$$

where Δ*F* is the decrease in the value of fluorescence expressed in arbitrary units, ΔK = *K*_FMN_-*K*_FAD_ is expressed as mM^−1^, V is the volume expressed in mL, *t* is time expressed in s and *m* is the protein amount in μmol. For activity measurements, the purified protein fraction (0.32 nmol) was incubated at 37 °C in 50 mM Tris/HCl, pH 7.5, containing 5 mM MgCl_2_, 2 μM FMN and 100 μM ATP, except where differently indicated in the legends to figures.

### 3.9. Measurements of FAD Cleavage Rate

The rate of FAD cleavage was measured by means of the differential fluorimetric properties of FAD with respect to FMN, essentially as in [15,27]. The purified protein fraction (0.32 nmol) was incubated at 37 °C in 50 mM Tris/HCl, pH 7.5, in the presence of 5 mM MgCl_2_ and of the substrates 0.5 μM FAD and 1 mM NaPPi, except where differently indicated in the legends to figures. The rate of FAD cleavage was expressed as μmol FAD · s^−1^ · μmol Δ_1-328_-hFADS^−1^, and was calculated from the rate of fluorescence increase, measured as the tangent to the initial part of the experimental curve by applying essentially the equation described in Section 3.8, except that Δ*F* was in this case the fluorescence increase expressed in arbitrary units.

### 3.10. Other Assays

Proteins were separated by SDS-PAGE (15% T polyacrylamide), according to Laemmli [28]. Quantitative evaluation of Coomassie Blue-stained protein bands was carried out using the Chemidoc imaging system equipped with the Quantity One software (Bio-Rad), as previously described [29].

In immunoblotting experiments, SDS-PAGE separated proteins were electro-transferred onto a PVDF membrane using a trans-blot semidry electrophoretic transfer cell (Sigma-Aldrich). The immobilized proteins were incubated over-night with a 3000-fold dilution of a polyclonal antiserum against the recombinant hFADS, as previously described in [14].

### 3.11. Kinetic Data Analysis

Data fitting was performed according to either the Michaelis–Menten equation:

$$v_{0}=V\text{max}\cdot s/(K\text{m}+s)$$

or allosteric kinetics equation:

$$v_{0}=V\text{max}\cdot s^{n}/(K\text{m}+s^{n})$$

Where

s0.5=√nKm

To fit the experimental data and to obtain estimates of the kinetic parameters, use was made of the GraFit software (Version 3.00, Erithacus Software LTd., Horlej, UK).

## 4. Conclusions

We presented here some features of the PAPS reductase domain, identified by bioinformatics and localized in the *C*-terminus half of human isoform 1 and 2 of FADS, which corresponds to residues 329–587 of hFADS1 (identical to residues 232–490 of hFADS2).

By the homology modeling strategy, we describe the structure of this domain and identify the protein motifs responsible for catalysis.

The polypeptide named Δ_1-328_-hFADS has been over-expressed in *E. coli* in its His-tagged form and purified as a FAD-binding protein performing an enzymatic catalytic activity, *per se*, not dissimilar from that of the entire enzyme.

Some regulatory features, such as cations sensitivity and inhibition constant for GTP of Δ_1-328_-hFADS, were changed with respect to the wild-type enzyme, thus suggesting a regulatory role for the lacking molybdopterin-binding domain, localized at the *N*-terminus of hFADSs.

## Figures and Tables

**Table I:** Comparison between the kinetic properties of Δ_1-328_-hFADS and 6-His-hFADS2. For the forward reaction (synthesis), *k*_cat_ was measured in the presence of 5 mM MgCl_2_, 100 μM ATP and 2 μM FMN. For the reverse reaction (pyrophosphorolysis), *K*_cat_ was measured in the presence of 5 mM MgCl_2_, 0.5 μM FAD and 1 mM NaPPi. *K*_m FMN_, *K*_m ATP_ and *K*_m NaPPi_; *K*_m FAD_ values were estimated in the presence of 100 μM ATP, 2 μM FMN, and 0.5 μM FAD, 1 mM NaPPi, respectively, by using the GRAFIT software (version 3.00, Erithacus Software LTd., Horlej, UK). Mg^2+^_50_, Co^2+^_50_ and Ca^2+^_50_ are the concentration of each metal required to reach 50% of the maximum activity.

	6-His-Δ_1-328_-hFADS (this study)	6-His-hFADS2 [13]
Forward reaction		
*k*_cat_ (s^−1^)	0.042 ± 0.001	0.069 ± 0.011
*K*_m FMN_ (μM)	0.24 ± 0.06	0.35 ± 0.08
*K*_m ATP_ (μM)	6.23 ± 1.5	15.3 ± 2.2
*K*_i GTP_ (mM)	7.5 (uncompetitive)	1.8 (uncompetitive)
Mg^2+^_50_ (mM)	0.05 ± 0.02	0.15 ±0.02
Co^2+^_50_ (mM)	0.08 ± 0.03	0.12 ± 0.02
Ca^2+^_50_ (mM)	0.10 ± 0.01	0.12 ±0.03
Reverse reaction		
*k*_cat_ (s^−1^)	0.0037 ± 0.0009	0.0052 ± 0.0001
*K*_m NaPPI_ (μM)	92.0 ± 0.5	82.1 ± 2.2
*K*_m FAD_ (μM)	n.d.	<0.1
Mg^2+^_50_ (mM)	0.20 ± 0.03	0.60 ± 0.08

n.d., not determined.

## Abbreviations

Rf: riboflavin
RFK: riboflavin kinase
FADS: FAD synthase or synthetase
FMNAT: FMN adenylyl transferase
hFADS: human FAD synthase
hFADS1: human FAD synthase isoform 1
hFADS2: human FAD synthase isoform 2
Δ_1-328_-hFADS: “truncated” form of hFADS lacking the *N*-terminal domain
PAPS: 3′-phosphoadenosine 5′-phosphosulfate
IPTG: isopropyl-thio-β-d-galactoside
